# Quince Seed and Flaxseed Mucilage as Natural Viscosity-Modifying Agents in Cementitious Composites: Rheological, Fresh-State and Mechanical Performance

**DOI:** 10.3390/polym18172135

**Published:** 2026-09-01

**Authors:** Ayşe Türk, Furkan Türk, Hayriye Nur Nayan, Çisem Kırbıyık Kurukavak, Ülkü Sultan Keskin

**Affiliations:** 1 Civil Engineering, Konya Technical University, Konya 42250, Turkey; 2 Chemical Engineering, Konya Technical University, Konya 42250, Turkey

**Keywords:** flaxseed, plant seed mucilage, quince seed, rheology, viscosity-modifying agent

## Abstract

This study evaluated the feasibility of using mucilage derived from plant seeds as a viscosity-modifying agent in cementitious composites. Mucilages derived from quince seeds (quince seed mucilage, QSM) and flax seeds (flaxseed mucilage, FSM)-which are readily available, low-cost, and sustainable sources of hydrocolloids-were dried and ground into powder, then used as viscosity-modifying agents in cementitious mixtures. Their effects on the mortars were compared with those of welan gum (WG), which is commercially used as a viscosity-modifying agent. According to the results, at a 0.1% dosage QSM and FSM reduced the spread diameter by 63% and 61% respectively, and significantly increased the V-funnel flow time, which exceeded 40 s. Rheological measurements revealed that all additives used increased viscosity, yield stress, and thixotropy. QSM increased plastic viscosity by approximately 8.9 times, and FSM by approximately 8.4 times. While the additives caused only a minor reduction in compressive strength (below 8%), they increased the resistance to segregation and extended the setting time. It was concluded that QSM and FSM can be used as viscosity-modifying agents in cement-based composites because they modulate the rheological properties without altering the mechanical properties of the mortars.

## 1. Introduction

As the range of applications for cement-based materials expands, the performance criteria required of these materials are also changing. Particularly in areas requiring more precise applications-such as self-compacting concrete, underwater concrete, and 3D-printable concrete-pure mechanical performance is no longer sufficient. In such specialized applications, fresh concrete must possess various rheological properties. Rheological parameters are of critical importance, particularly in 3D-printable concrete applications, which have become increasingly widespread in recent times and remain an active area of research. In such applications, viscosity and yield stress must be carefully balanced to prevent segregation. High viscosity makes the application difficult, while low viscosity leads to problems such as bleeding and segregation. Additionally, fresh concrete must possess high thixotropy to support itself and the layers above it. Therefore, balanced rheology is of critical importance in specialized concrete applications in terms of workability and concrete quality.

In cement-based composites, the balance of rheological parameters can generally be achieved using viscosity-modifying agents (VMA). Viscosity-modifying agents are used in cement-based systems to increase cohesion, yield stress, plastic viscosity, and stability, and to reduce leaching and bleeding [1,2,3]. Also known as rheology-modifying additives, consist of water-soluble polymers. The long-chain molecules of VMAs bind to water molecules, adsorbing and retaining a portion of the mixing water; this increases the mixture’s yield stress and plastic viscosity while reducing bleeding [4,5]. In cement-based systems, VMAs include welan gum [6,7,8,9], xanthan gum [10], diutan gum [3,4], and starch ether [11], which are among the additives currently in widespread use. Welan gum, diutan gum, and xanthan gum are natural gums based on high-molecular-weight polysaccharides derived from microbial sources and produced via aerobic fermentation [3,4,12].

Welan gum is a microbial polysaccharide produced by fermentation and used in cement systems [13,14]. Reported studies on process optimisation show that welan gum yields can range from about 0.53 to 0.76 g/g, with concentrations from roughly 22.7 to 41.0 g/L depending on strain and fermentation strategy [15,16,17,18]. Because many studies focus on improving yield and reducing substrate costs, production economics remain an important research topic for microbial polysaccharides [17,18]. In this context, exploring plant-derived mucilages such as quince seed and flaxseed may be a reasonable direction, particularly if they can provide viscosity-modifying functionality while relying on comparatively low-cost and renewable raw materials. Growing pressure to reduce the construction industry’s carbon footprint has led researchers to seek lower-cost, renewable, and environmentally friendly alternatives.

Due to the ever-increasing economic and environmental costs, the construction sector now demands lower-cost and environmentally friendly building materials [19]. Current viscosity-modifying agents can be effective, but their cost and limited sustainability motivate the search for alternative materials that can provide comparable rheological control in cement-based systems. Plant-seed mucilages are reported to be sustainable, low-cost, biodegradable and non-toxic biopolymers [20,21,22,23], which makes them attractive candidates as bio-based viscosity-modifying agents. This study investigates the feasibility of using natural plant seed mucilages as viscosity-modifying agents (VMAs) in cement-based composites. Seed mucilage is a hydrophilic polysaccharide-rich layer released upon hydration and is associated with water retention and seed hydration functions [24]. Their hydroxyl- and carboxyl-containing polysaccharide structures can increase water retention and contribute to gel-like structuring, viscosity, and cohesion in aqueous systems [25,26]. In cement-based materials, polysaccharide-based VMAs are already known to increase cohesion and viscosity, thereby helping to reduce bleeding and segregation in highly flowable mixtures [1,8,27,28].

When placed in water, quince seeds release a mucilage composed mainly of water-soluble polysaccharides, commonly referred to as quince seed mucilage or extract [29]. Recent reviews describe quince seed mucilage as a promising hydrocolloid with thickening, viscoelastic, and stabilizing potential, especially because its composition is responsive to environmental and processing conditions [25,29]. Likewise, flaxseed mucilage is widely recognized for its water-binding, thickening, and gel-forming capacity, which explains its use as a stabilizing hydrocolloid in different industries [30,31]. Given these functional properties, quince and flaxseed mucilages provide a plausible bio-based basis for evaluating natural VMA alternatives in cementitious systems, although their performance must be demonstrated experimentally within the cement matrix rather than inferred directly from food or pharmaceutical applications [32,33].

Although both flaxseed mucilage and welan gum are recognized as modifiers of cement rheology, no controlled study has yet compared their effects directly with those of quince seed mucilage under equivalent conditions, particularly with respect to fresh-state rheology and hardened-state performance. Moreover, while quince seed mucilage appears to be a promising hydrocolloid, its behavior in cementitious systems remains insufficiently investigated. Accordingly, assessments of cement performance continue to depend largely on inferences drawn from the existing literature on rheology and composition. It still largely relies on inferences from the literature regarding its rheology and composition.

Accordingly, this study examines plant-based viscosity-modifying agents obtained by aqueous extraction of mucilage from quince seeds and flaxseeds, followed by drying and grinding. By focusing on these two seed-derived biopolymers as VMA candidates, the study addresses the need for renewable and potentially cost-effective alternatives to conventional admixtures while also testing whether they can deliver the rheological stability required in cement-based composites. Their effects on segregation resistance, rheology, and mechanical properties of cement-based composites are then compared with a commercial reference admixture. In this way, the study aims not only to assess performance, but also to clarify the practical potential and distinct contribution of quince seed and flaxseed mucilages as bio-based VMAs.

## 2. Materials and Methods

The aim of this study was to compare VMAs derived from quince seeds and flaxseeds-as alternatives to commercial VMAs-with the commercial product Welan gum. The Welan gum used in the study was in fine powder form (95% of particles < 75 µm) and had a specific gravity of 1.45 g/cm^3^. To evaluate quince seed and flaxseed mucilage as VMAs in cement-based composites, mortars containing various proportions of VMA and mortars without VMA (control) were prepared. The segregation resistance of the produced mortars, the flow parameters (V-funnel, spread), the compressive strengths of the hardened mortars, and the viscosity and thixotropic properties of the cement pastes were investigated.

### 2.1. Materials

#### 2.1.1. Binder and Other Materials

CEM I 42.5 R Portland cement (Konya Çimento Sanayii A.Ş. Turkey), compliant with the EN 197-1 standard [34], was used as the binder in this study. CEN standard sand (Limak Çimento, Merkez, Turkey), in accordance with the EN 196-1 standard, was used in the preparation of mortar samples. Tap water was used as mixing water. The rheological behaviour and retardation of cementitious systems are strongly influenced by the molecular characteristics of the superplasticiser, such as its molecular weight and side-chain structure, which govern its adsorption on cement particles [35,36]. To ensure that the observed differences arose solely from the viscosity-modifying agents, the same superplasticiser (Sika ViscoCrete (Sika Yapı Kimyasalları A.Ş., İstanbul, Turkey)) was used at a constant dosage in all mixtures. Dried quince (*Cydonia oblonga*) seeds and flax (*Linum usitatissimum*) seeds, purchased from the market, were used as the source of mucilage; the seeds were cleaned of foreign matter prior to extraction.

#### 2.1.2. Mucilage Extraction

**Figure 1:**
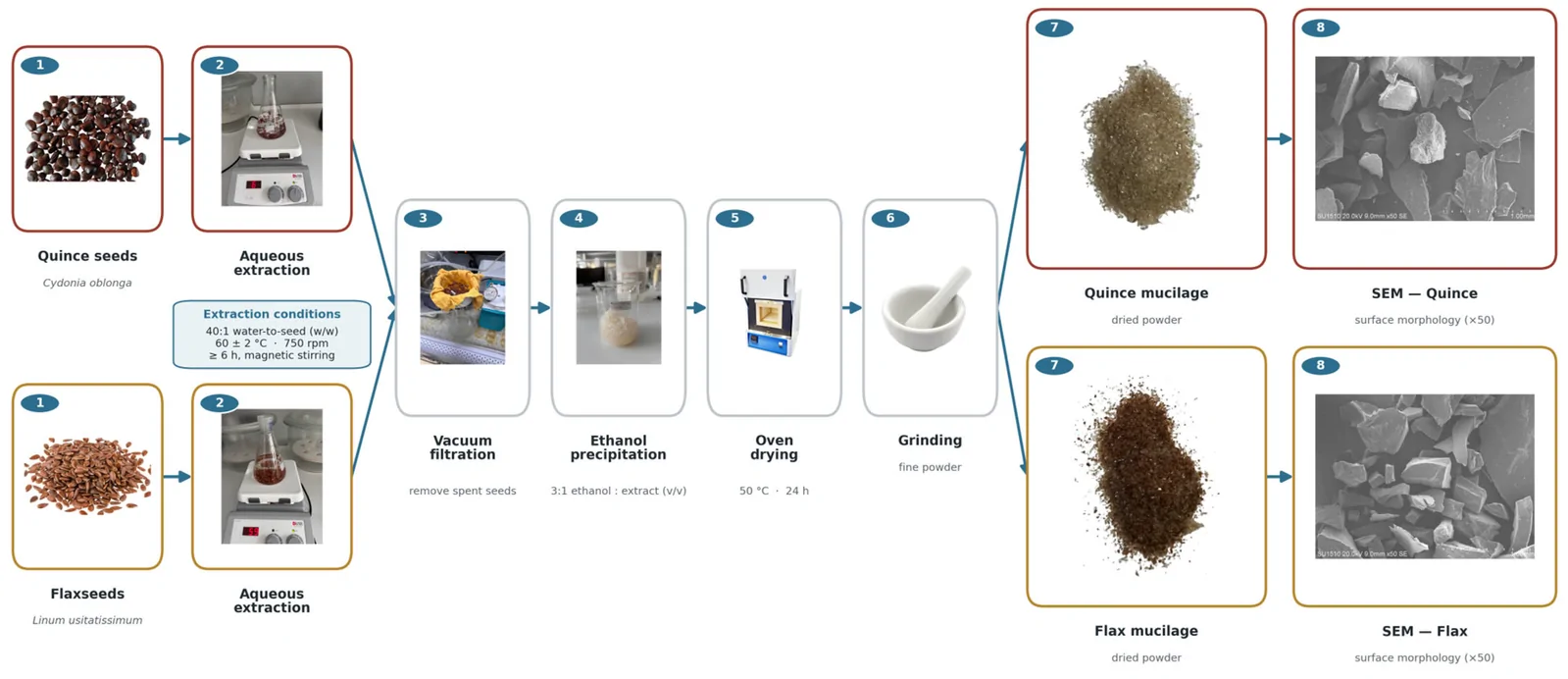
Extraction of mucilage from quince and flaxseed.

The traditional and cost-effective solvent extraction method, which is the most commonly preferred in the literature for obtaining quince seed and flaxseed mucilages, was used. Mucilage extraction from plant seeds was performed using an aqueous extraction method, with parameters determined based on optimization results reported in the literature [37]. Water extraction is also widely used industrially to isolate functional polysaccharides from seeds. For this purpose, the moisture contents of quince seeds and flaxseeds were first determined to be 5–7% and 6–8%, respectively, according to the AOAC standard procedure. Mucilage was then extracted from the plant seeds using a distilled water-to-seed ratio of 40:1 (weight/weight). The mixture of seeds and distilled water was heated to 60 ± 2 °C and stirred at 750 rpm for at least 6 h using a magnetic stirrer with a heating plate (DLAB, Beijing, China). After vacuum filtration (Büchner funnel and vacuum pump, sourced locally in Turkey), the mixture was precipitated to achieve an ethanol-to-extract ratio of 3:1. Finally, the extracted mucilage was dried in an oven at 50 °C for 24 h and then ground. A simple schematic representation of the extraction of quince seed and flaxseed mucilage is shown in Figure 1.

The extraction yields of the dried mucilage samples were calculated according to Equation (1).

(1) $$E_{y}=\frac{W_{1}}{W_{2}}\times 100\;$$

*E_y_*: extraction yield, %.

*W*_1_: dry extract weight, g.

*W*_2_: seed weight, g.

It was determined that the mucilage yield of quince seeds was 13%, while that of flaxseeds was 7%. As expected, quince seeds have a higher mucilage yield than flaxseeds in terms of dried mucilage content, and these results are consistent with the literature [38].

**Figure 2:**
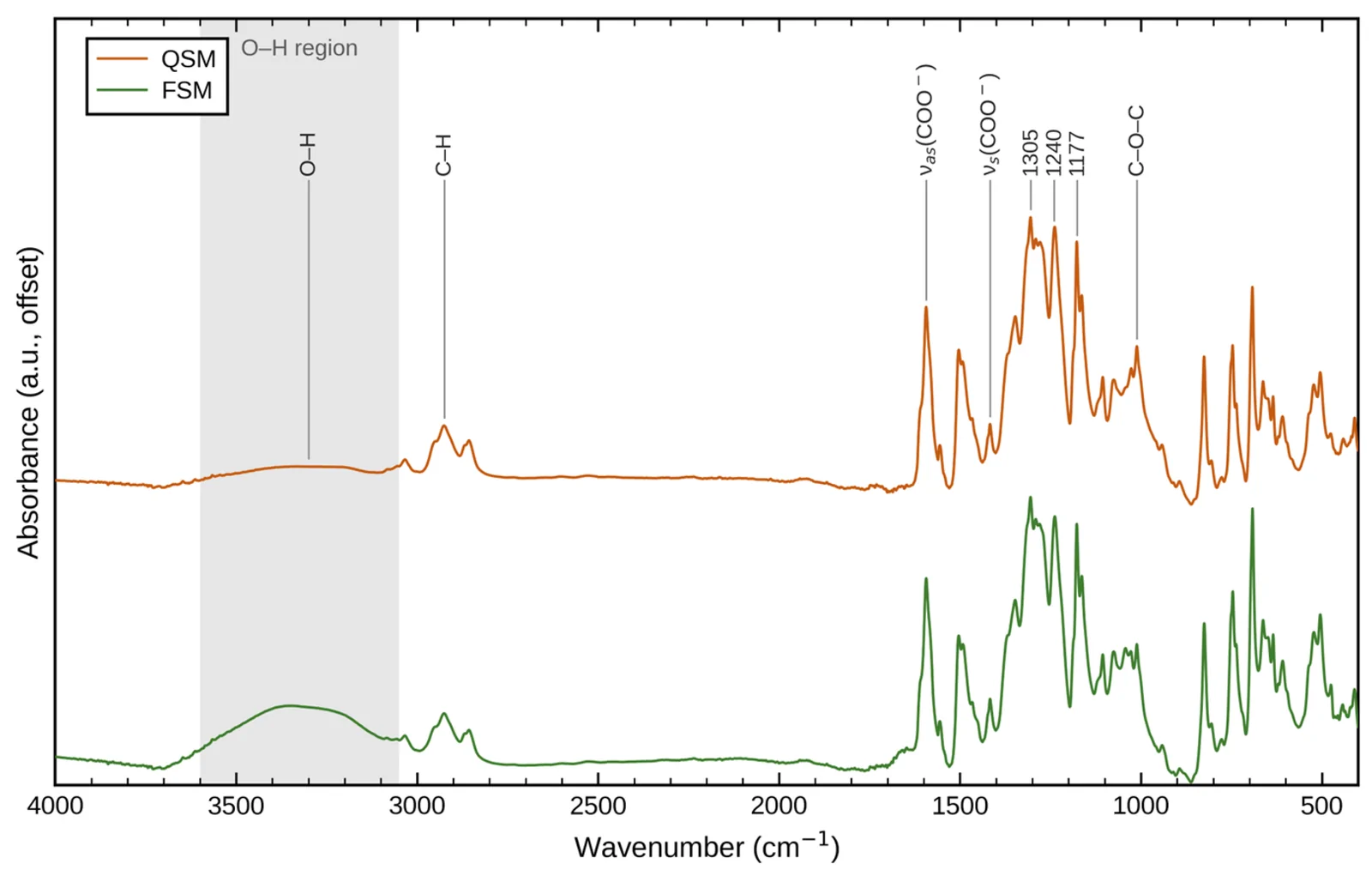
FTIR analyses of QSM and FSM.

The FTIR spectra of QSM and FSM are presented in Figure 2. In both spectra, the broad absorption between 3600 and 3050 cm^−1^ corresponds to O-H stretching, and the bands at 2925 and 2856 cm^−1^ to C-H stretching of the sugar units [39]. The strong band at 1593 cm^−1^, together with its counterpart near 1417 cm^−1^, is assigned to the asymmetric and symmetric stretching of carboxylate groups, indicating the presence of uronic acid residues in both polysaccharides. The bands between 1305 and 1011 cm^−1^ arise from C-O and C-O-C stretching of the pyranose rings and glycosidic linkages, with the principal skeletal band at 1011 cm^−1^; absorptions in the 610–1251 cm^−1^ region are associated with the β-D-glycosidic bonds of uronic acid in the polysaccharide structures [40]. The absorption pattern in both cases is characteristic of pectinaceous polysaccharides and confirms that the mucilages were successfully isolated from the seeds.

The two spectra were further compared quantitatively (Table 1). All resolved bands coincide to within 1 cm^−1^, that is, within the spectral resolution of the measurement, indicating that the two mucilages contain the same functional groups in the same chemical environments. Because absolute band intensities are not directly comparable between separately measured specimens, peak height ratios within each spectrum were used for comparison. The ratio of the asymmetric carboxylate band at 1593 cm^−1^ to the skeletal C-O-C band at 1011 cm^−1^ was 2.01 ± 0.41 for QSM and 2.22 ± 0.54 for FSM, which are similar within the uncertainty introduced by the choice of baseline, indicating comparable carboxylate content relative to the polysaccharide backbone. The only substantial difference between the two spectra lies in the O-H stretching region, where the ratio A(O-H)/A(1011) was 0.13 ± 0.02 for QSM and 0.66 ± 0.14 for FSM. This difference persisted across all baseline configurations examined and is therefore considered robust. It should be noted, however, that the O-H region is also sensitive to adsorbed moisture, so this difference is not attributed solely to the structural hydroxyl content of the polymers.

These observations indicate that the two mucilages are not distinguishable by infrared spectroscopy in terms of the functional groups governing their interaction with the cement matrix, and in particular that their carboxylate contents relative to the polysaccharide backbone are comparable. The difference in rheological behaviour reported in the Results section is therefore unlikely to originate from the functional group composition. Other factors not resolved by infrared spectroscopy, such as molecular weight, chain conformation and degree of branching, may instead be responsible, and their determination is recommended for future work.

#### 2.1.3. Preparation of Mixtures

Within the scope of this study, cement mortars were prepared using the mixing ratios given in Table 2. In the literature, the usage rates of various viscosity-modifying agents (welan gum, diutan gum, cellulose-based admixtures, etc.) are frequently chosen to be between 0% and 0.25% [4,7,8,41,42]. Generally, using additives within this range provides sufficient viscosity improvement. Therefore, the additive ratio to be used in this study was selected to be between 0.005% and 0.1% by weight of the binder. All three admixtures were dosed on an equal-mass basis by weight of cement, in accordance with standard practice for chemical admixtures, to allow direct comparison of their effectiveness at replacing a commercial VMA.

**Figure 3:**
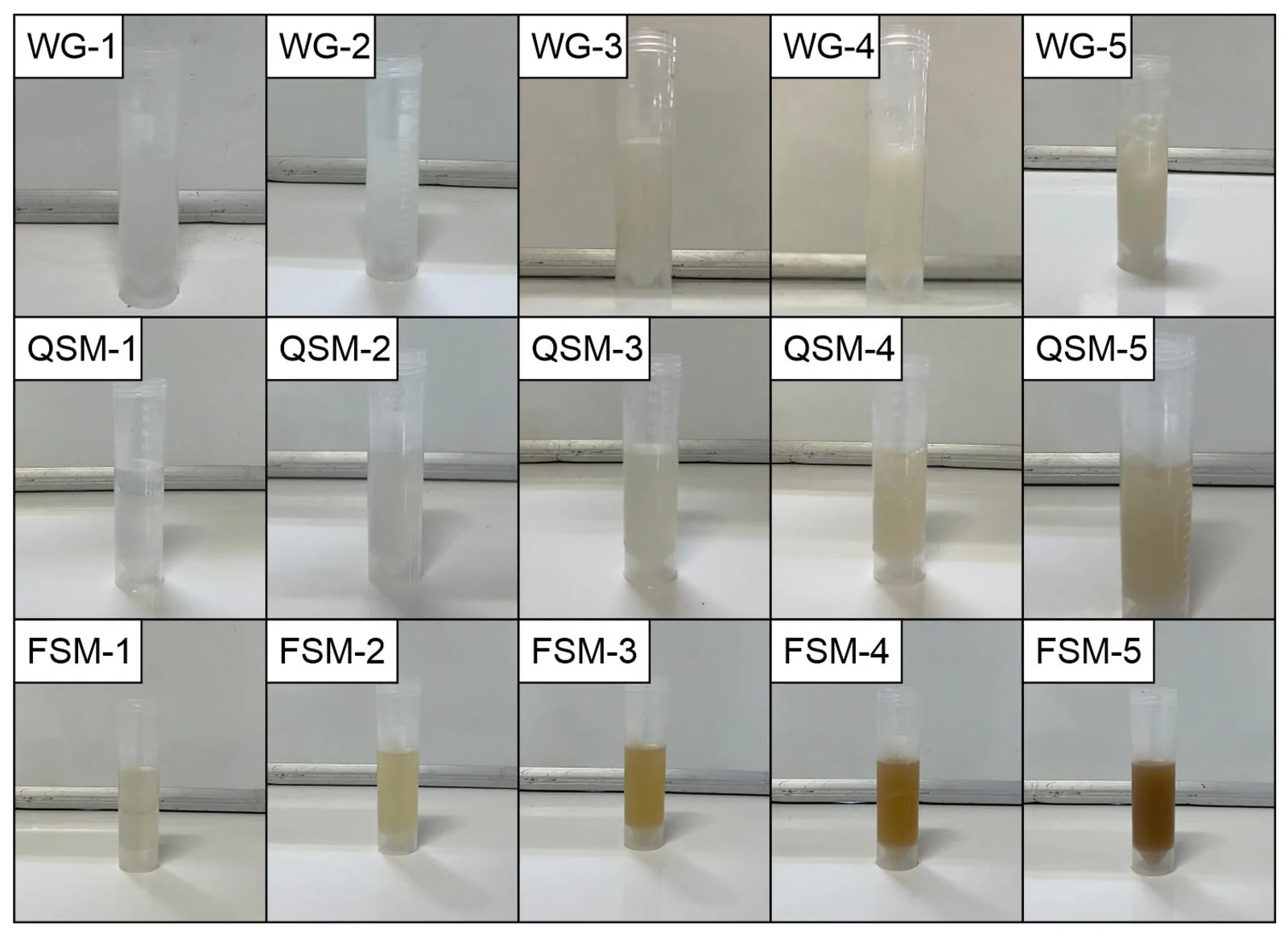
Aqueous solutions of additives.

A portion of the mixture solution was set aside, the additive to be used (WG, QSM, FSM) was added to it, and the mixture was stirred in an ultrasonic homogenizer (Bandelin HD 2070, Berlin, Germany) for 1 h, resulting in the WG, QSM, and FSM solutions, as shown in Figure 3.

### 2.2. Method

#### 2.2.1. Fresh-State Properties

**Figure 4:**
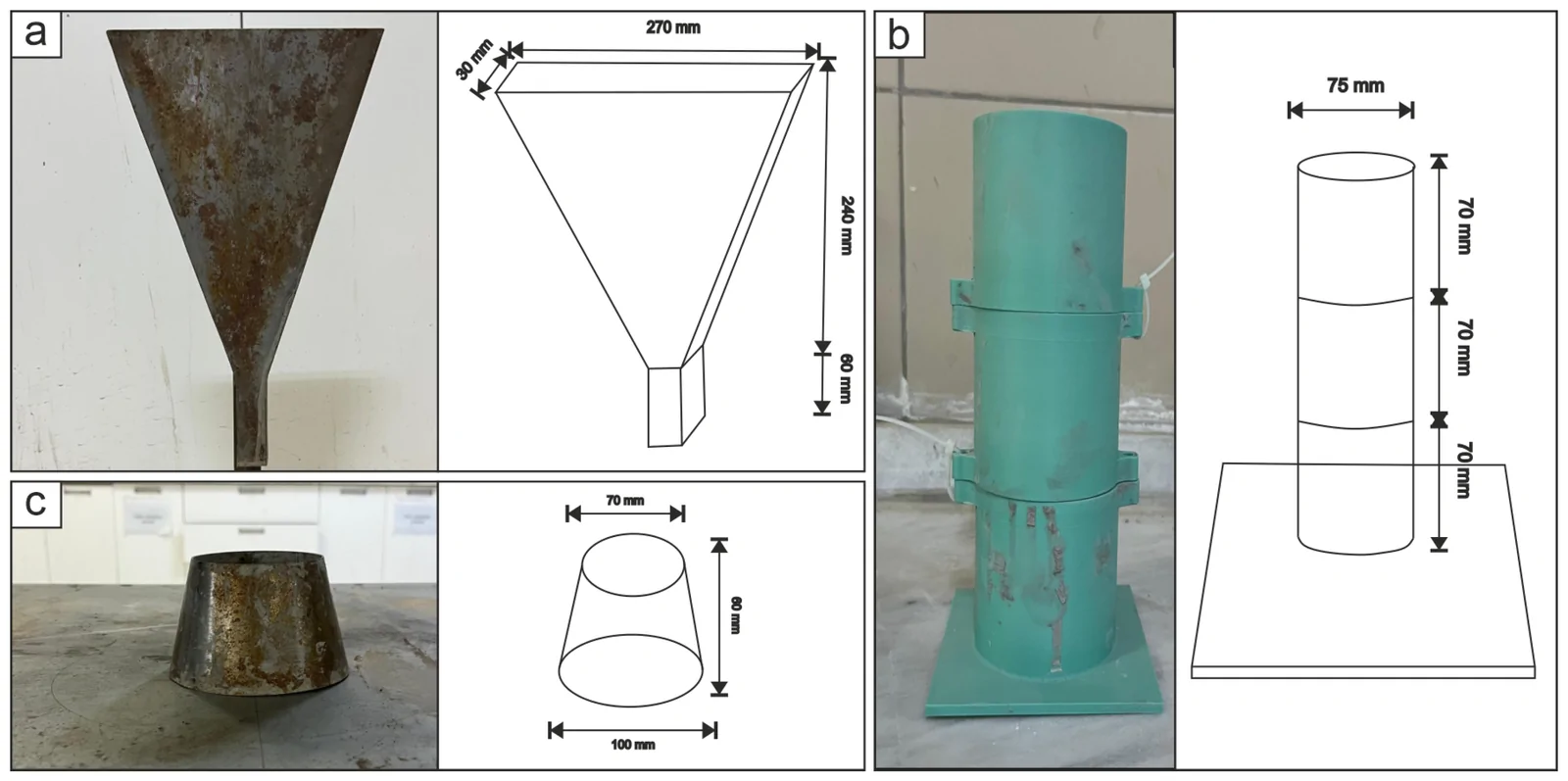
Test apparatus: (**a**) mini V-funnel, (**b**) segregation test, (**c**) mini slump.

The deformability of the prepared mortars was determined using the mini-slump test, and their flow times were determined using the mini-V-funnel test. Resistance to static segregation was assessed using the mini-column segregation test, a mortar-scale adaptation of the column technique described in ASTM C1610/C1610M-21 [43], developed for self-consolidating mortars by Libre et al. [44] and Mehdipour et al. [45] and subsequently validated against the standard method by Mahdikhani and Ramezanianpour [46]. The apparatus consisted of a PVC column of 75 mm internal diameter and 210 mm height, divided into three separable sections of 70 mm each. The column was filled with fresh mortar in a single lift without compaction and left undisturbed for 15 min, after which the sections were separated and the material from the uppermost and lowermost sections was recovered. Each portion was washed over a 300 µm (No. 50) sieve, and the retained fine aggregate was oven-dried and weighed. The resistance to segregation of the mortars was calculated according to Equation (2). All fresh-state tests were carried out in triplicate. The reported values represent the mean of three measurements, and the error bars in the figures denote one standard deviation. The fresh-state property test apparatus used in the study is shown in Figure 4.

(2) $$SI=2\left[\frac{Ab-At}{Ab+At}\right]\times 100$$

*SI*: static segregation index, %.

*At*: aggregate mass in the upper section of the column, g.

*Ab*: aggregate mass in the lower section of the column, g.

#### 2.2.2. Rheology

**Figure 5:**
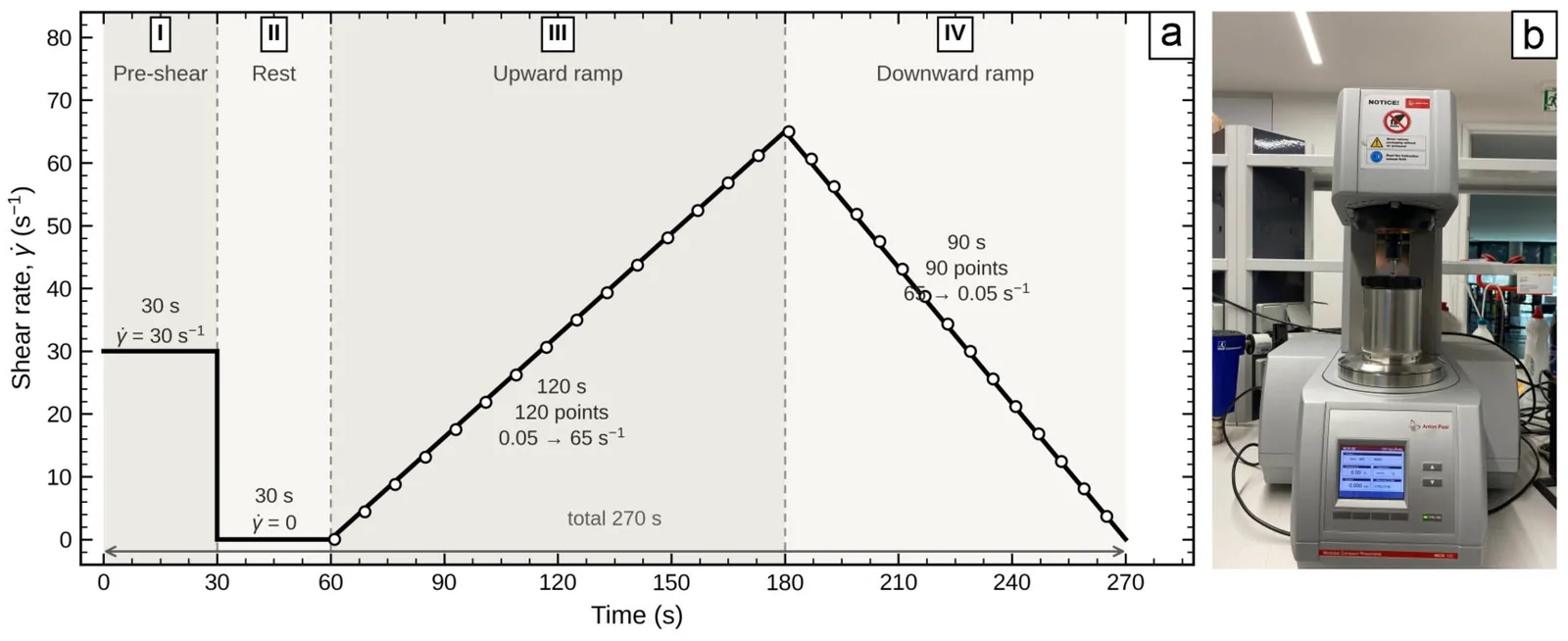
Rheological measurement setup and test protocol: (**a**) shear rate profile applied to the cement paste, (**b**) rheometer.

Rheological measurements were carried out on the cement pastes, whose flow behaviour governs that of the corresponding mortars. A rotational rheometer (Anton Paar MCR 102, Graz, Austria) equipped with a Building Material Cell (BMC) was used. The cell comprises a cylindrical cup of 74 mm internal diameter and 95 mm height fitted with a removable anti-slip cage, which prevents the sample from sliding along the inner wall, and a two-bladed open-frame stirrer as the rotating geometry, in the standard configuration supplied by the manufacturer. All measurements were performed at ambient laboratory temperature (23 ± 2 °C). For the rheological measurements, each prepared sample was first mixed for 30 s at a shear rate of 30 s^−1^; subsequently, the shear rate was first increased to 65 s^−1^ and then reduced again. Shear stress values were recorded during the periods of increase and decrease in shear rate. The measuring configuration and the applied shear profile are presented in Figure 5.

The Bingham model given in Equation (3) was used to determine the plastic viscosity. In addition, the Herschel–Bulkley model (Equation (4)) was fitted to the flow curves, as it describes the non-linear behaviour of the pastes more accurately. The yield stress was obtained from the Herschel–Bulkley model and the plastic viscosity from the Bingham model, since the Herschel–Bulkley model has no direct plastic-viscosity equivalent. Both models were fitted to the descending branch of the flow curve. The thixotropic properties of the mortars were determined using Equation (5), which represents the area between the rising and falling curves of the cement paste.

(3) $$\tau =\tau_{0}+{µ}_{p}\times \gamma$$

*τ*: shear stress, Pa.

*τ*_0_: yield stress, Pa.

*µ_p_*: plastic viscosity, Pa.s.

γ: shear rate, s^−1^.

(4) $$\tau =\tau_{0}+K\times \gamma$$

*τ*: shear stress, Pa.

*τ*_0_: yield stress, Pa.

*K*: consistency index, Pa.s*^n^*.

γ: shear rate, s^−1^.

(5) $$A=\int_{\gamma \min}^{\gamma \max}\eta_{1}\gamma d\gamma \;-\;\int_{\gamma \min}^{\gamma \max}\eta_{2}\gamma d\gamma$$

*A*: area of the hysteresis loop.

γ: shear rate.

*η*_1_: up curve.

*η*_2_: down curve.

γmin: 0.05, s^−1^.

γmax: 65, s^−1^.

#### 2.2.3. Normal Consistency and Setting Time

To assess the effects of the additives used on setting times, the normal consistencies of the mixtures containing the highest additive content were determined, and the initial and final setting times were established in accordance with TS EN 196-3 [47].

#### 2.2.4. Compressive Strength

To determine the effect of additive use on mechanical performance, the compressive strengths of 28-day mortar specimens, produced in dimensions of 40 × 40 × 160 mm in accordance with TS EN 196-1 [48], were determined.

## 3. Results and Discussion

### 3.1. Fresh-State Properties

**Figure 6:**
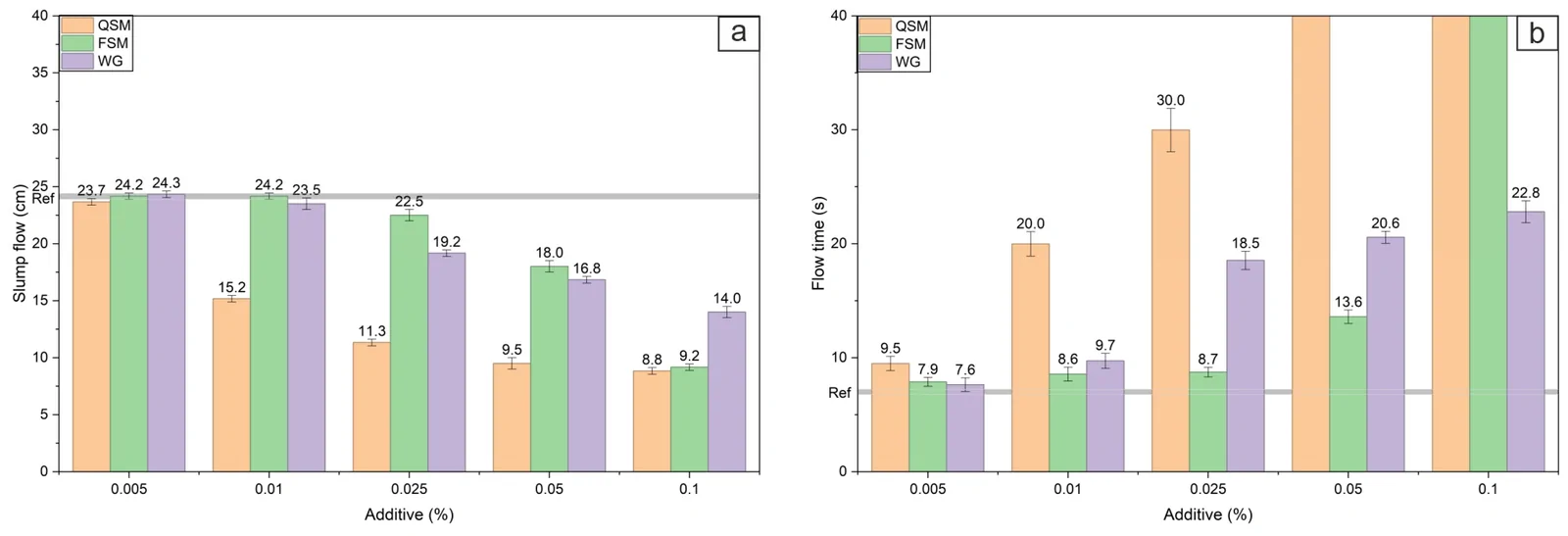
Test results: (**a**) slump flow, (**b**) flow time.

The results of the mini-slump and mini-V-funnel tests for fresh mortar mixtures are presented in Figure 6a. The results show that, regardless of the type of admixture, the spread diameter of the mortars decreases as the admixture dosage increases. Furthermore, among the admixtures used, QSM is the most sensitive to increases in dosage. Although QSM and FSM caused a similar reduction in the spread diameter at the highest additive dosage (0.1%), within the 0.005–0.025% dosage range, FSM maintained the spread value of the reference sample; QSM, on the other hand, caused a dramatic reduction in the spread diameter even at a dosage of 0.01%. The WG additive, on the other hand, resulted in a more controlled and smaller reduction in spread diameter. At the highest additive dosage, the spread diameter decreased by 63% for QSM, 61% for FSM, and 41% for WG compared to the reference.

The flow times of the fresh mortar mixtures, measured using the mini-V-funnel test, are presented in Figure 6b. All three additives increased the flow time compared to the reference. When QSM was added at concentrations of 0.05% and 0.1%, and FSM at 0.1%, the flow time exceeded 40 s, so the test was stopped at the 40th second. Similar to the spread diameter results, QSM was found to be the most sensitive to dosage increases among the additives used. Even when added at a concentration of 0.01%, QSM increased the flow time by a factor of 2.8. FSM and WG, on the other hand, showed a more gradual increase. However, FSM exhibited a sudden increase at the highest dosage, exceeding the 40 s experiment duration. WG was the additive with the least effect on flow time among the additives tested, and when used at the highest dosage, it increased the flow time by a factor of 3.2.

**Figure 7:**
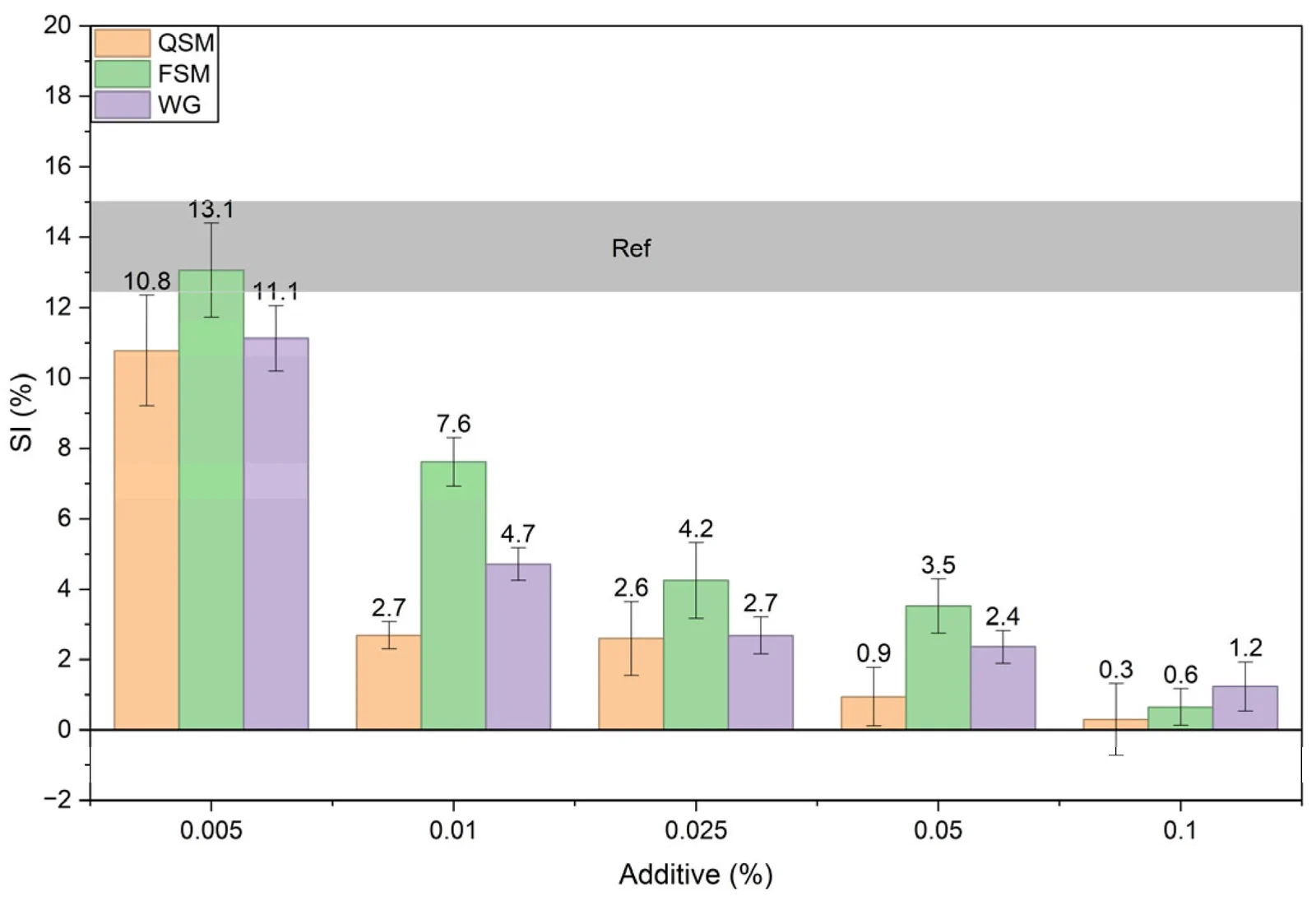
Segregation resistance test results.

Figure 7 shows the segregation index calculated from the segregation test results. According to the data obtained, the segregation index of the reference sample was calculated to be 14.73 ± 0.29%. It was found that the mortar produced without additives was prone to segregation. However, all viscosity-modifying agents used in this study reduced the segregation index, depending on the dosage. Similar to the results of other tests, WG exhibited more controlled behavior. However, the QSM and FSM additives exhibited more dramatic behavior, similar to the results of the mini-V-funnel test. This indicates that QSM and FSM behave in a more cohesive manner compared to WG. These findings are consistent with the results of the rheological and consistency tests.

WG is a viscosity-modifying agent that has been extensively studied in the literature. The mechanism by which WG in solution forms three-dimensional networks and causes macromolecules to entangle with one another as their concentration increases is responsible for the rise in viscosity [49,50]. It is also known that WG prevents segregation in cement-based composites by increasing the matrix viscosity and yield stress [8,41]. This increase results in a loss of spread in the mini-slump test and an increase in flow time in the mini-V-funnel test. In this study as well, WG gradually reduced the spread diameter and increased the flow time in a dose-dependent manner. It exhibited more controlled behavior compared to the other additives used in the study. Similar to the spread diameter and flow time tests, WG was also observed to gradually reduce the segregation of the mortar in the segregation tests. Furthermore, the rheological measurement results also corroborate these test results for the WG-containing samples.

Since mucilages are materials with moisture-retaining properties, they can adsorb water. As mucilages dissolve in water, the polysaccharide chains unfold, allowing for ionization. As a result of this ionization, anionic groups bind to cations in the cement, and flocculation occurs due to these interactions between cement particles and the mucilage [51]. Thanks to the resulting physical bonds, chain entanglements, and water-retention properties, the spread diameters of the mixtures decrease, while V-funnel flow times increase.

The mucilages used in this study affected both the spread and flow times of the mixtures. As the additive usage rate increased, a decrease in spread diameter was observed, while an increase in flow times was noted. Similarly, rheological measurement results confirm this behavior. However, the spread and flow time tests differ slightly from the rheological results. For example, mini-V-funnel test results could not be obtained for mixtures containing 0.05% QSM, 0.1% QSM, and 0.1% FSM due to the absence of flow. In this case, it is initially expected that the viscosities of these mixtures would be higher than those of the other mixtures. However, the cohesion factor should not be overlooked here. As reported in the literature, cohesion is a separate rheological parameter, even though it is related to viscosity and yield stress. The mini-V-funnel test measures the flow time of the mixture. Therefore, this test is influenced by both cohesion and viscosity. The cohesion factor can cause low viscosity to result in a longer flow time than high viscosity [52,53,54]. This study demonstrates that cement pastes can exhibit different flow times (see Figure 6b) even though they have similar viscosities (0.05% QSM ≈ 1.23 Pa·s, 0.05% FSM ≈ 1.04 Pa·s, as shown later in Section 3.2). Furthermore, the segregation test results clearly show that, among mixtures with similar viscosity and yield stresses, the more cohesive one may exhibit higher resistance to segregation. Taking a V-funnel flow time exceeding the 40 s measurement limit together with a flow spread below 10 cm as the threshold of practical placeability, the effective working range was 0.005–0.025% for QSM and 0.025–0.05% for FSM, whereas WG remained placeable across the entire range studied. At 0.005%, the flow spread of all three mixtures was within 0.5 cm of the reference, indicating that the onset of effective viscosity-modifying action lies close to this dosage.

### 3.2. Rheology

**Figure 8:**
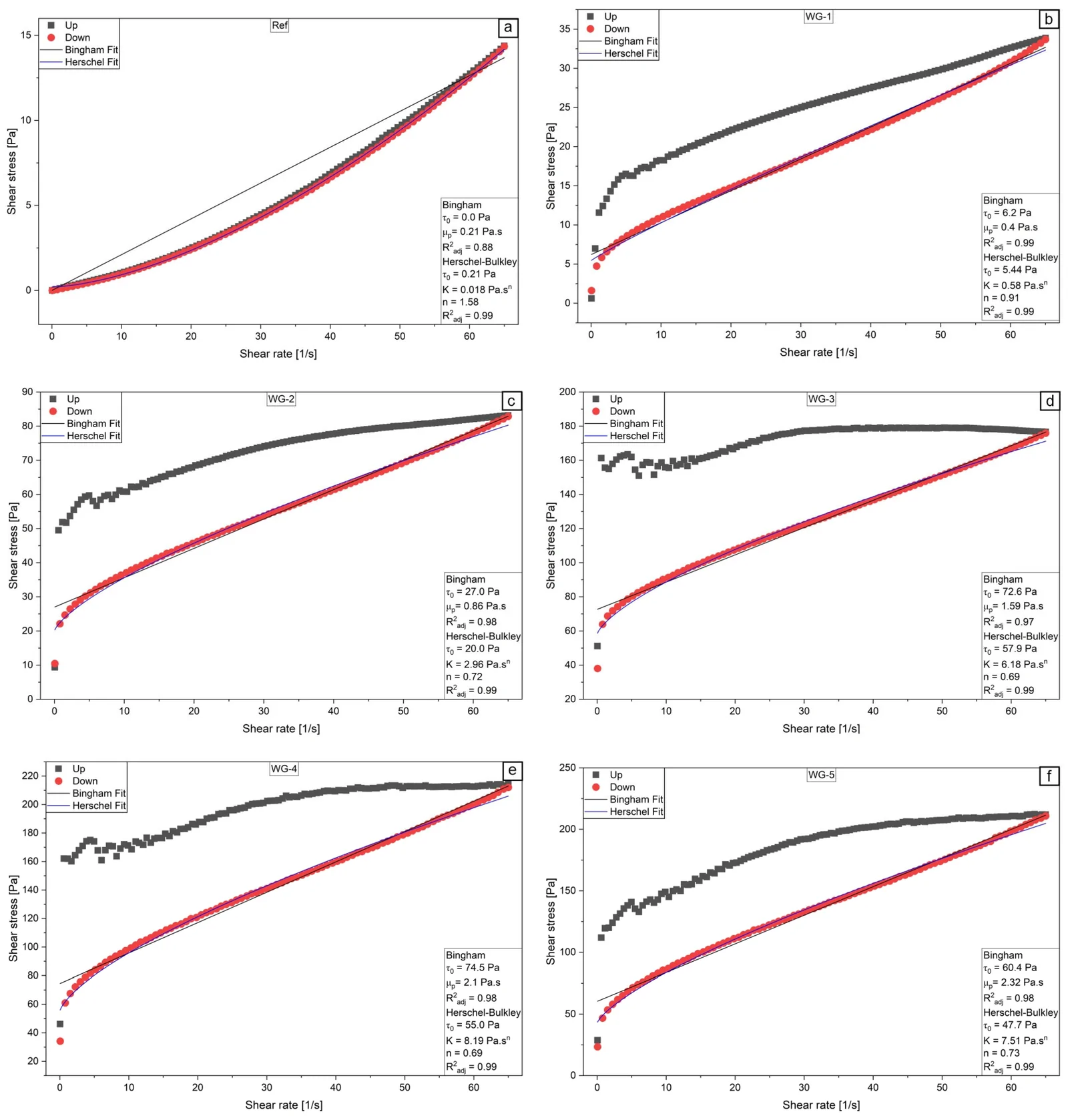
Hysteresis loops of cement pastes with reference and WG additives: (**a**) Reference, (**b**) 0.005% WG, (**c**) 0.01% WG, (**d**) 0.025% WG, (**e**) 0.05% WG, (**f**) 0.1% WG.

**Figure 9:**
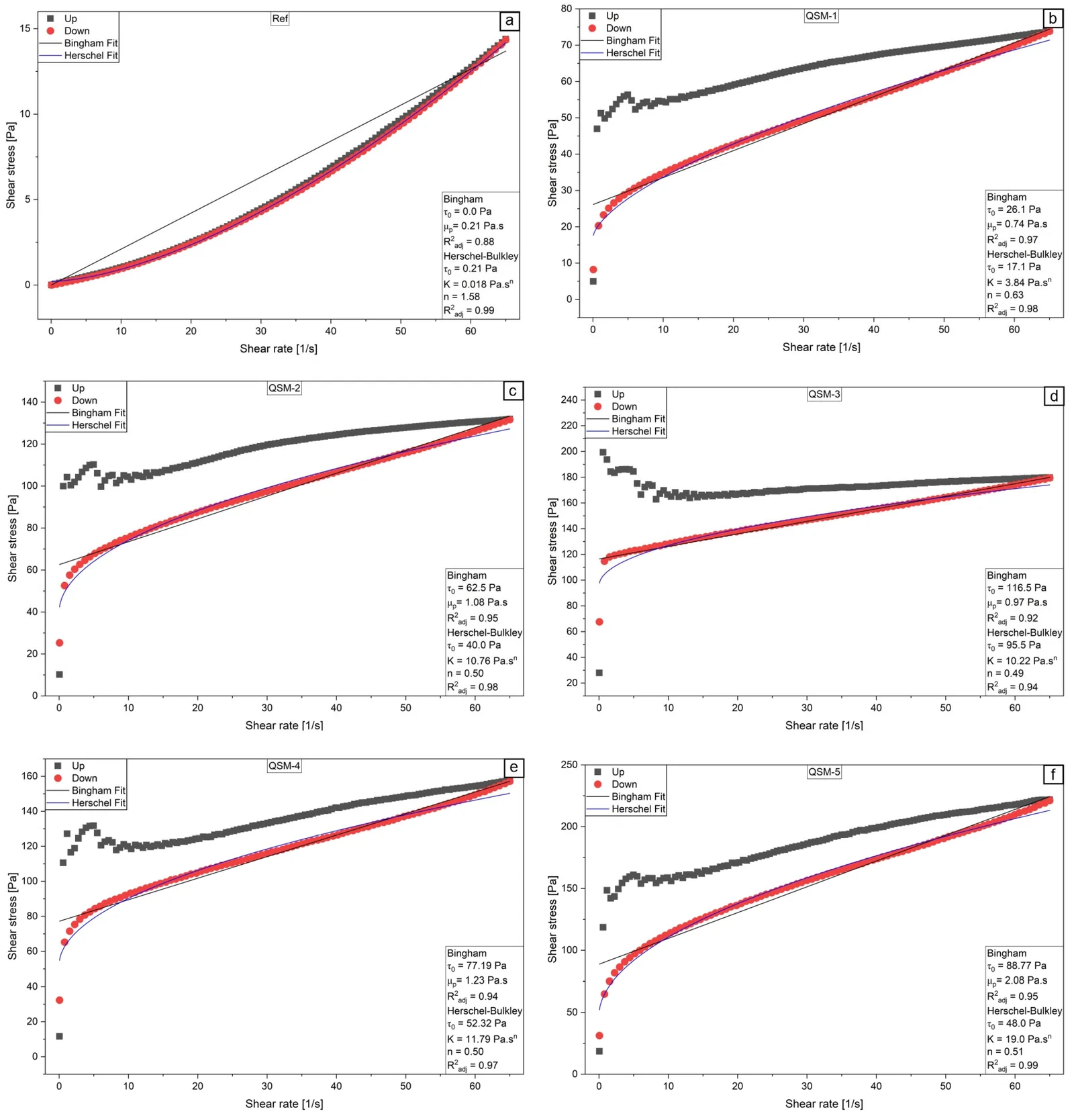
Hysteresis loops of cement pastes with reference and QSM additives: (**a**) Reference, (**b**) 0.005% QSM, (**c**) 0.01% QSM, (**d**) 0.025% QSM, (**e**) 0.05% QSM, (**f**) 0.1% QSM.

**Figure 10:**
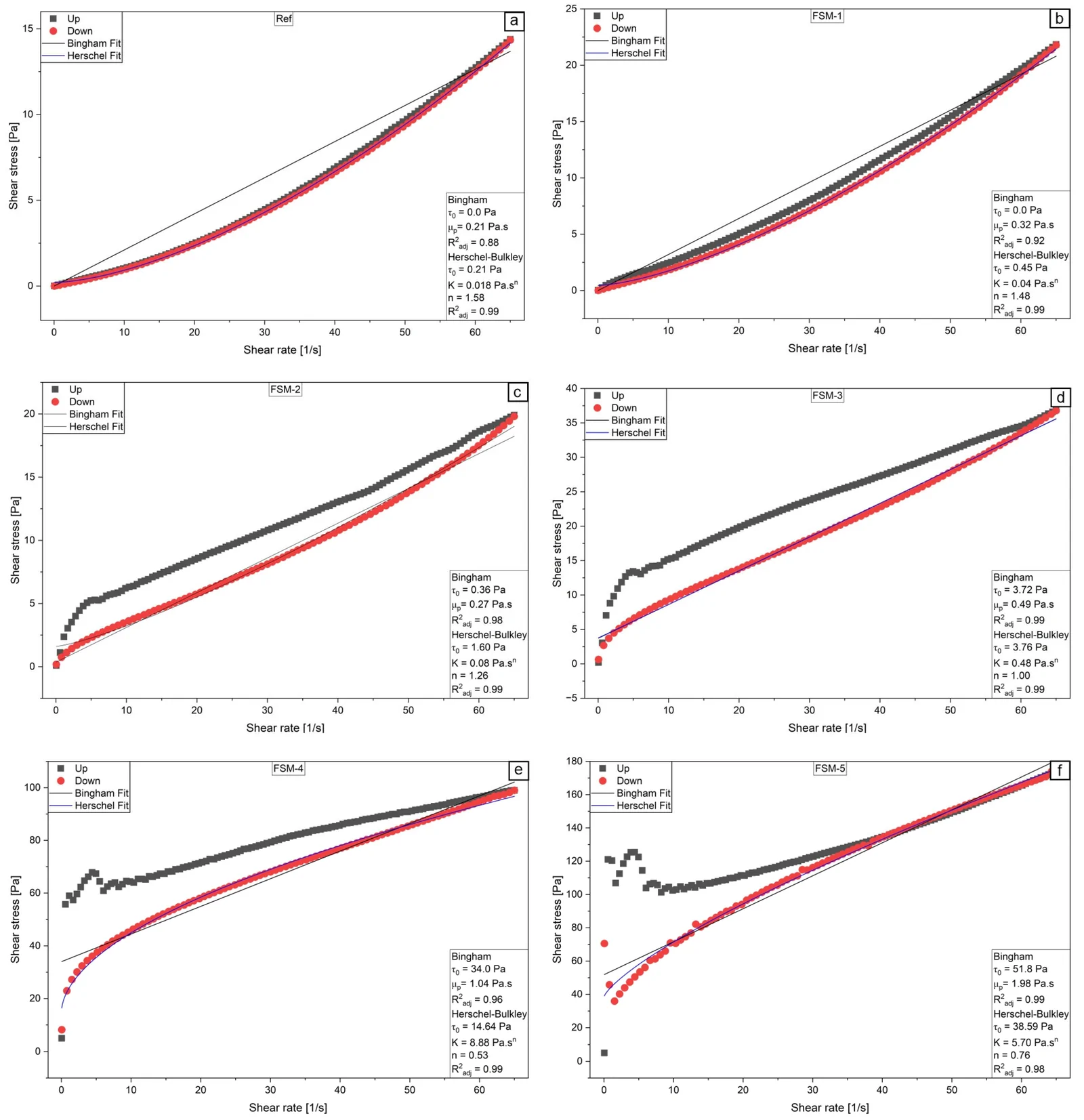
Hysteresis loops of cement pastes with reference and FSM additives: (**a**) Reference, (**b**) 0.005% FSM, (**c**) 0.01% FSM, (**d**) 0.025% FSM, (**e**) 0.05% FSM, (**f**) 0.1% FSM.

**Figure 11:**
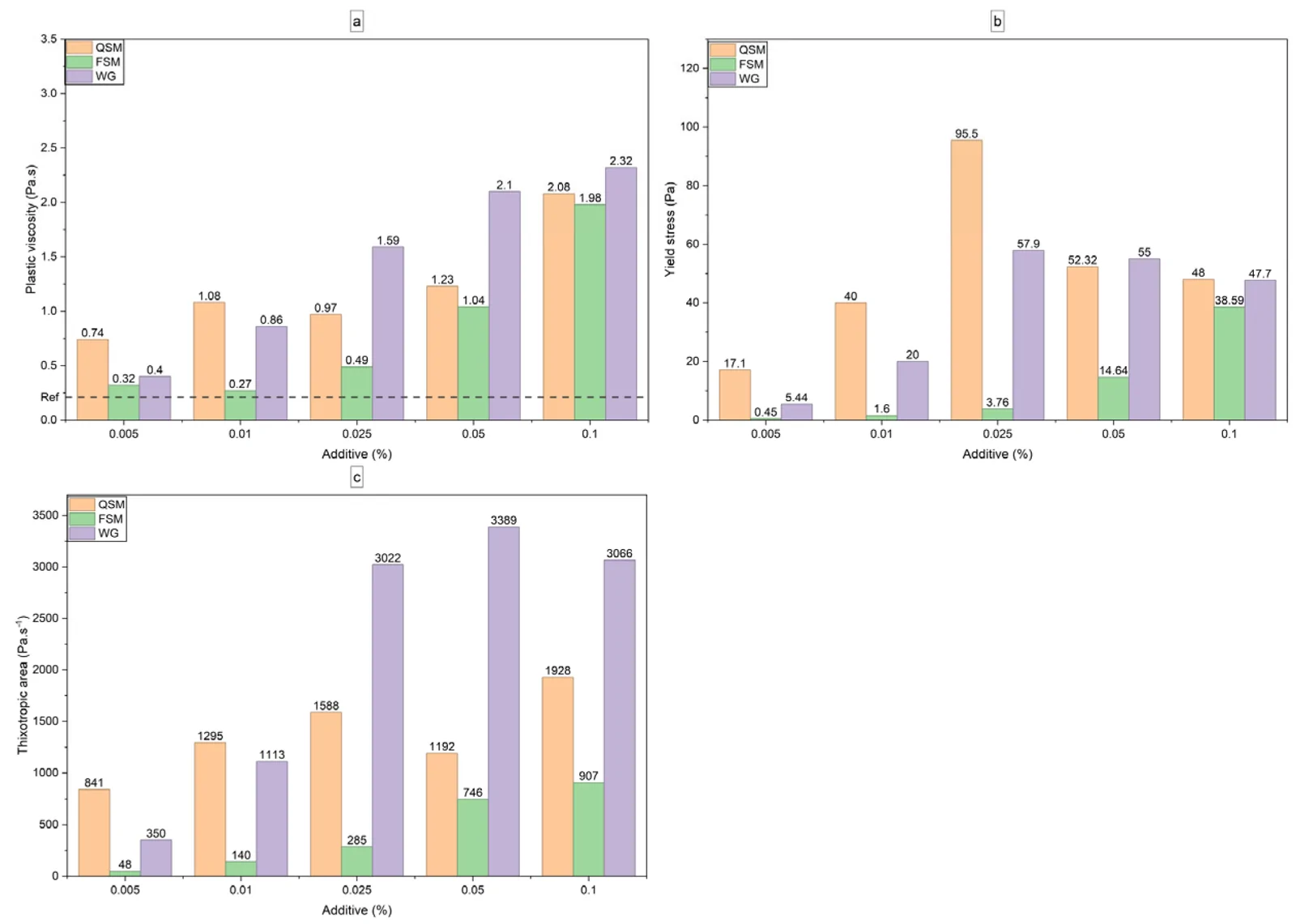
Rheological parameters: (**a**) plastic viscosity, (**b**) yield stress, (**c**) thixotropic area.

Based on the results of rheological tests on cement pastes, the plastic viscosity, yield stress, and thixotropic area of the mixtures were calculated. The hysteresis loops obtained from rheometer measurements on cement pastes containing WG are shown in Figure 8, samples containing QSM in Figure 9, and samples containing FSM in Figure 10. The Bingham and Herschel–Bulkley parameters obtained for all mixtures are summarised in Table 3. The values of plastic viscosity, yield stress, and thixotropic area are presented in Figure 11.

As can be seen from flow-curve models and Figure 11, it was observed that the additives increased the plastic viscosity compared to the reference mixture. The additives increased viscosity in a dose-dependent manner. WG was found to be the most effective additive on plastic viscosity. When WG was used at a concentration of 0.1%, it increased the plastic viscosity by a factor of 10. It was observed that the QSM additive increased viscosity in a dose-dependent manner. When QSM was added at a concentration of 0.1%, it increased viscosity by approximately 8.9 times. Although the FSM additive increased viscosity in a dose-dependent manner, it was the additive that produced the smallest increase in viscosity. When used at a concentration of 0.1%, it increased viscosity by 8.4 times. The fact that the additives increased viscosity supports the results of the mini-V funnel and mini-slump tests. The yield stress obtained from the H-B model of the reference mixture was determined to be 0.21 Pa. It was observed that QSM was the additive that increased the yield stress the most. When QSM was used at a rate of 0.025%, the yield stress was 95.5 Pa. After QSM, the additive that increased the yield stress the most was WG. When WG was used at a rate of 0.025%, the yield stress was calculated to be 57.9 Pa. The yield stress of QSM peaked at 0.025% before decreasing at higher dosages. This non-monotonic trend originates from an irregular, plateau-like response of the 0.025% mixture at low shear rates, associated with shear localisation in this highly structured paste, which raises the fitted yield stress and lowers the goodness-of-fit (adjusted R^2^ = 0.94). The plastic viscosity, determined from the high-shear region, increased monotonically with dosage and provides a more reliable indication of the overall thickening behaviour. The yield stress of this mixture is therefore interpreted with caution. It was observed that FSM had the least effect on the yield stress. When FSM was used at a concentration of 0.1%, the yield stress was 38.59 Pa. It was observed that, regardless of the type of additive, the thixotropic range of the additive-containing samples increased gradually with increasing dosage. Similar to the results for plastic viscosity, WG was found to be the most effective additive in increasing the thixotropic area. The yield stress did not increase uniformly with dosage for all admixtures. For QSM, the value at 0.025% was elevated owing to an irregular low-shear response of this mixture (adjusted R^2^ = 0.94), associated with shear localisation, and is interpreted with caution. For WG, the yield stress decreased gradually above 0.05% while the plastic viscosity continued to increase and the fit remained excellent (R^2^ = 0.99); this decoupling is consistent with an overdosing effect at high polymer content, where the excess polymer weakens the interparticle network while continuing to thicken the liquid phase.

It was observed that the rheological measurement results were consistent with one another and in line with the literature. Rheological properties such as yield stress and plastic viscosity are related to the fresh-state spread and flow time characteristics of mortars [55]. For example, it is known that there is a strong inverse relationship between yield stress and the spread diameter in the mini-slump test. The increase in yield stress associated with higher additive ratios in cement pastes may be attributed to the additive materials’ hydrophilic structures and their ability to adsorb mixing water due to their high water-retention capacity [56,57]. Water molecules adsorb onto the surface of WG-a long-chain, high-molecular-weight microbial polysaccharide-and cannot drain freely. Consequently, they move with the WG during flow, thereby increasing plastic viscosity and yield stress [1,49]. Additionally, WG, which is anionic, can adsorb onto cement particles [58]. At high concentrations, WG can cause long polymer chains to entangle with one another and form a robust network at low shear rates [28,41]. This network formed by the entanglement of polymer chains is interpreted as the cause of increased thixotropy [59,60]. In this study as well, rheological measurements demonstrated that the addition of WG improves the viscosity, yield stress, and thixotropic properties of cement-based mixtures.

The results obtained from this study for QSM and FSM are highly consistent with those reported in the literature. Studies examining the rheological properties of QSM and FSM report that these mucilages possess high gelling properties [61,62,63,64]. The intertwining of polysaccharide chains forms a network, leading to an increase in viscosity. This network formed by polysaccharide chains can be disrupted by high shear forces, and the network structure can reform over time after shear ceases; thus, quince seed and flaxseed mucilages exhibit thixotropic behavior [64]. In this study as well, rheological measurements demonstrated that the addition of QSM and FSM improved the viscosity, yield stress, and thixotropic properties of cement-based mixtures.

### 3.3. Normal Consistency and Setting Time

**Figure 12:**
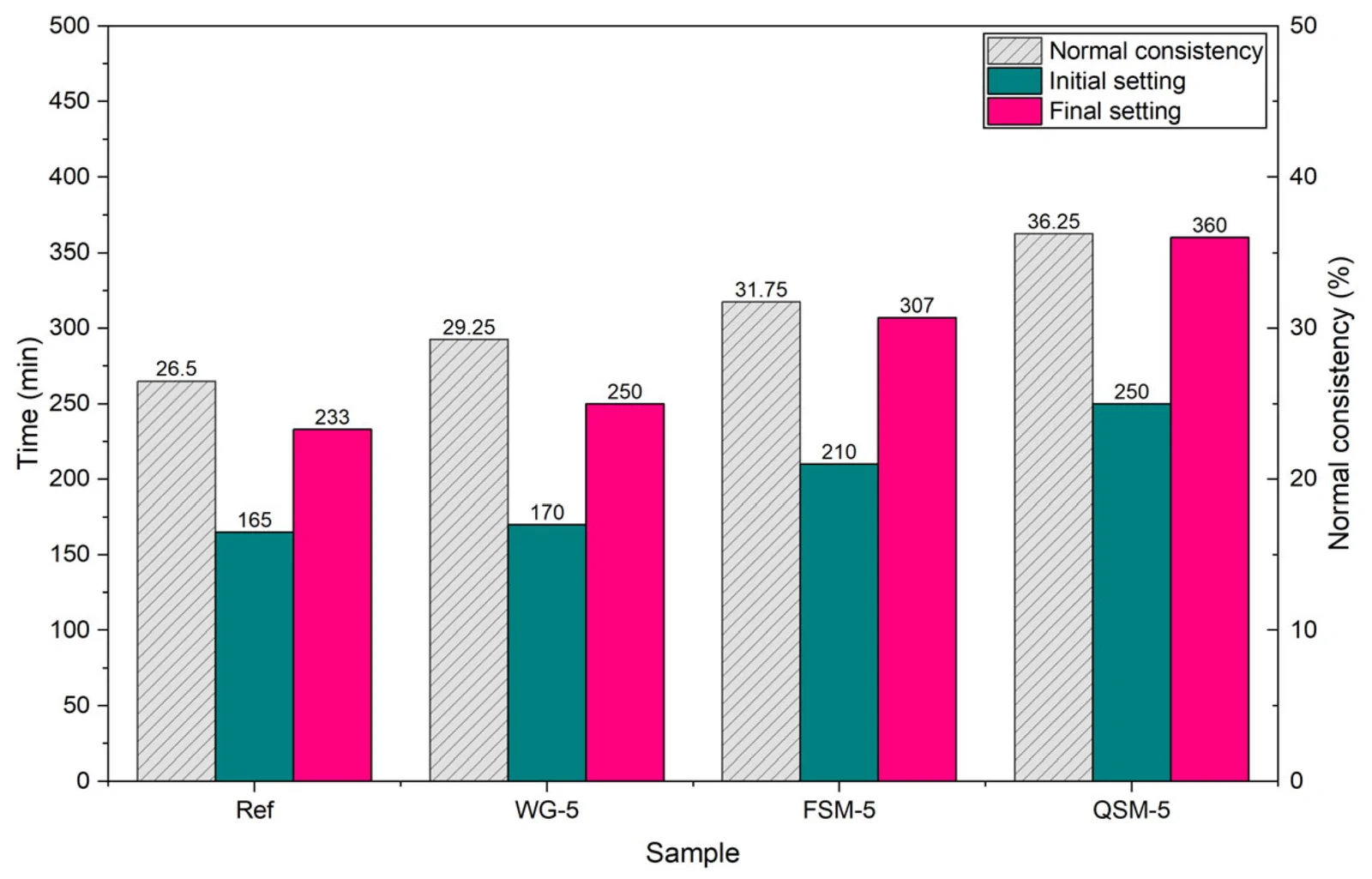
Setting time and normal consistency of samples.

As part of this study, the normal consistency and setting times were determined for a reference sample containing no admixture and for samples containing admixtures at a rate of 0.1%. The findings are presented in Figure 12. It is clearly evident from Figure 12 that, regardless of the type of admixture, all admixtures increased the amount of water required for normal consistency. While the amount of water required for normal consistency in the reference mixture was 26.5%, this value reached 36.25% in the QSM sample. It is a common finding that products with a hydrophilic structure [65] increase the amount of water required for normal consistency. The effectiveness of the additives in increasing normal consistency was determined to be WG, FSM, and QSM, respectively, in accordance with the rheological and consistency tests. In parallel with the normal consistency tests, increases in the initial and final setting times were observed. Similarly, the effectiveness of the additives in prolonging the setting time was determined to be WG, FSM, and QSM, respectively, in accordance with the rheological and consistency tests. Other studies in the literature report that mucilages obtained from various sources and WG prolong the setting time by forming hydrate-retarding films [33,66]. It is understood that the mechanisms causing increases in viscosity and yield stress also affect the normal consistency.

### 3.4. Compressive Strength

**Figure 13:**
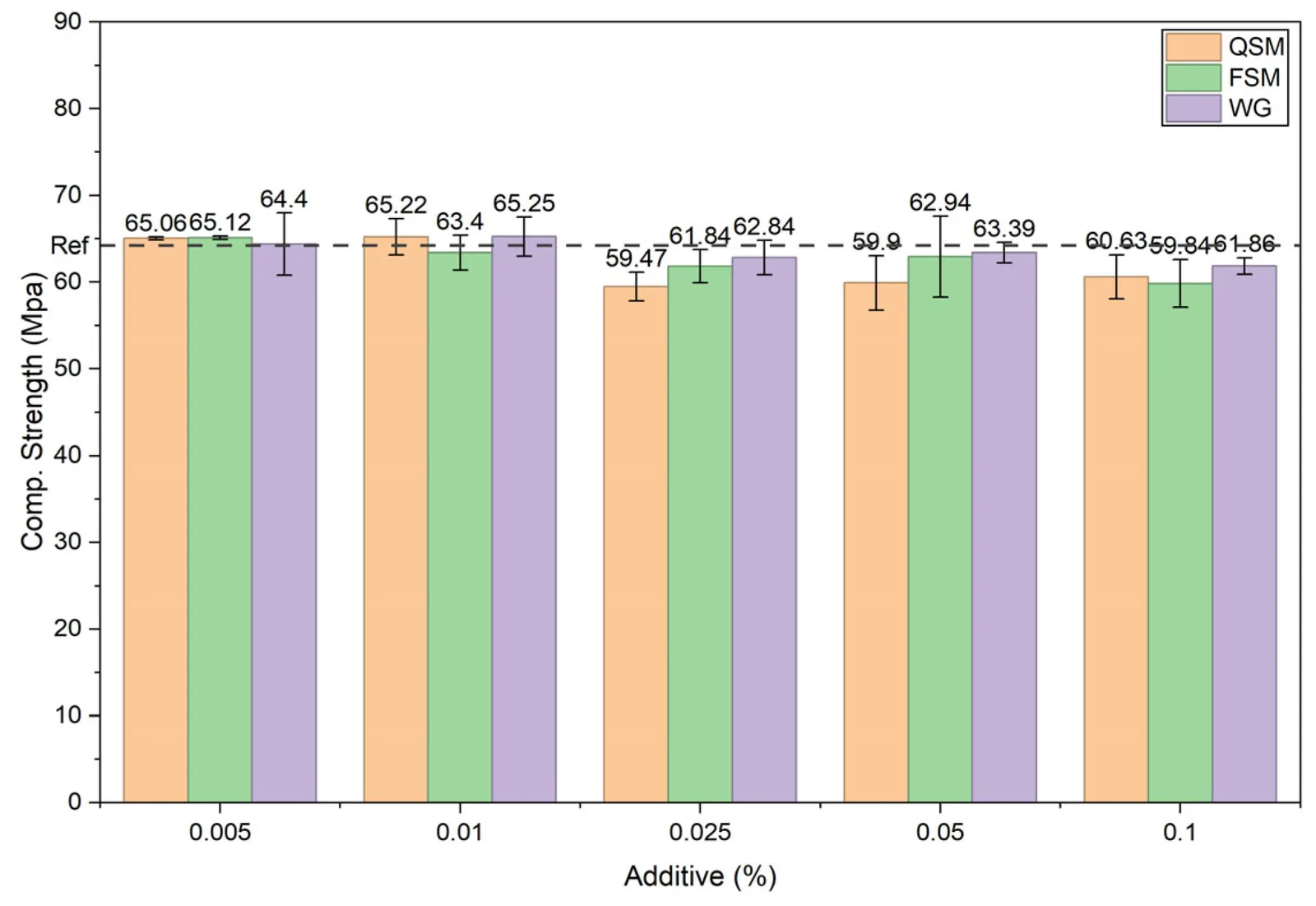
The 28-day compressive strength results.

The results of the 28-day compressive strength test on the specimens are presented in Figure 13. The results show that, particularly at low additive usage rates (≤0.01%), the additives do not cause a significant change in compressive strength. However, at higher additive usage rates (≥0.025%), a loss in compressive strength of approximately 8% was observed. No statistically significant reduction was detected for most mixtures; however, given the limited number of replicates, this absence of a detected effect should not be taken as evidence that no reduction occurred. Compressive strength ranged from 59.5 to 65.3 MPa across all mixtures. One-way analysis of variance indicated a significant effect of dosage for QSM (F(4,10) = 5.17, *p* = 0.016), whereas no significant dosage effect was found for FSM (*p* = 0.264) or WG (*p* = 0.412). Pairwise comparison of individual mixtures against the reference yielded no differences that remained significant after correction for multiple comparisons. Given the replicate number, the present design has approximately 80% power to detect differences of about 6 MPa (10% of the reference strength); the observed reductions, which did not exceed 8%, therefore lie close to the resolution limit of the test programme.

The literature reports that mucilage additives derived from various plant sources may be beneficial in cement-based composites up to a certain concentration. It is believed that mucilages, which act as internal curing agents by retaining water due to their polar structure, promote the formation of a dense matrix, ensure a prolonged and healthy hydration process, and reduce CH precipitation [33,67]. Additionally, the fact that samples with mucilage additives exhibit low porosity, combined with the dense matrix formed, may result in higher strength compared to the reference sample. However, the extent of the resulting strength increase is debatable. Furthermore, the strength increase becomes more pronounced at later ages. Ultimately, in this study, viscosity-modifying agents were not used to increase the strength of cement-based composites. As a general rule, the fact that additives used in cement-based composites for purposes other than strength enhancement do not have a negative effect on strength demonstrates the suitability of the additive used.

A similar mechanism applies to WG. While WG used in cement-based composites at high dosages may cause a decrease in strength due to delayed hydration at early ages, it can exhibit a slight increase in strength at lower dosages and at later ages [42,68].

### 3.5. Microstructure

**Figure 14:**
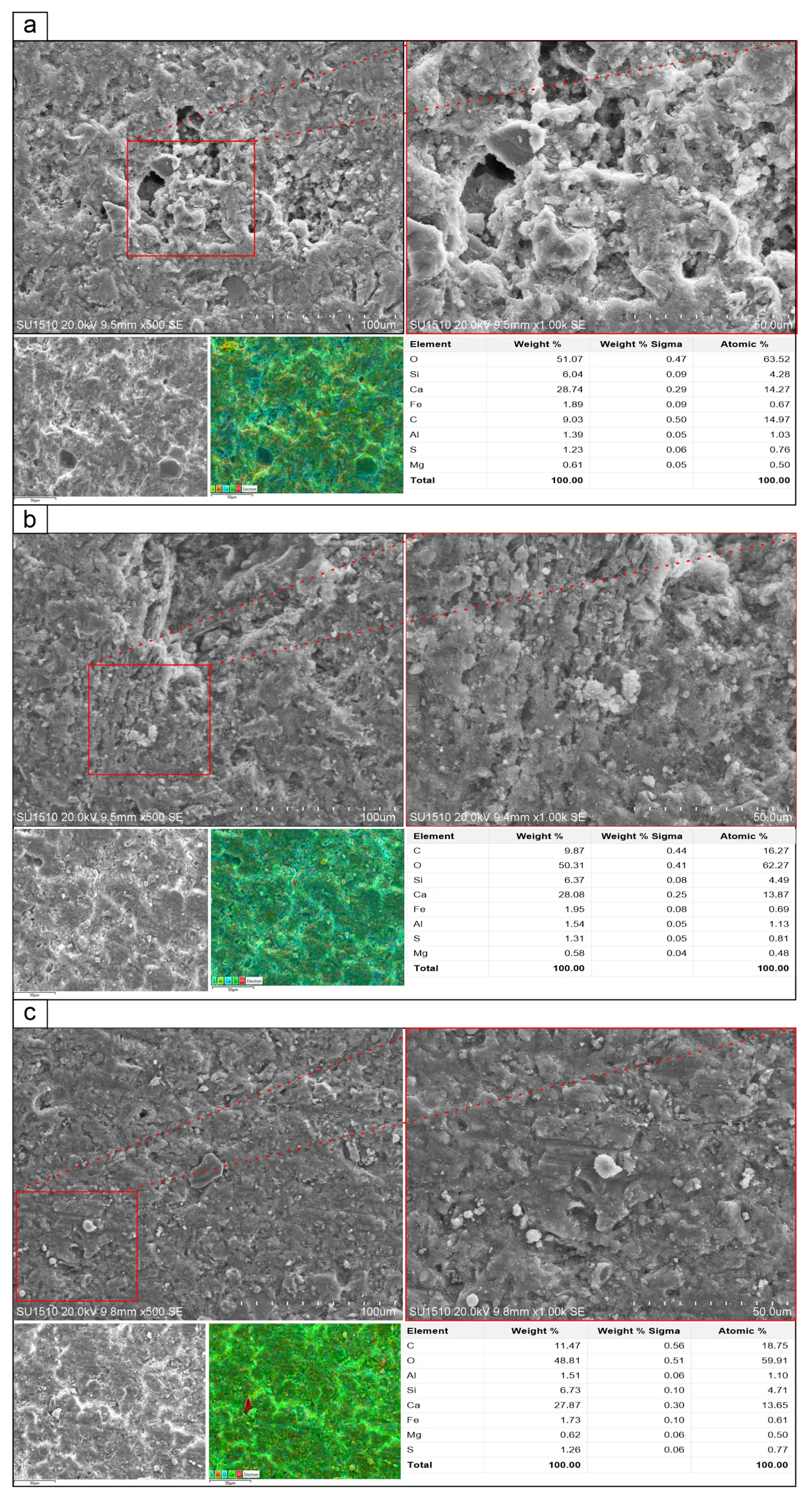
SEM-EDS analysis: (**a**) Ref, (**b**) QSM-5, (**c**) FSM-5.

Figure 14 shows the SEM-EDS analysis results for the reference, QSM-5, and FSM-5 samples. It should be emphasised at the outset that all phase-related interpretations in this section are provisional. They are based on the morphological and elemental information provided by SEM-EDS, which is indirect and semi-quantitative and cannot identify crystalline hydration phases. The assignments of C-S-H and portlandite, and the inferred interactions between the mucilages and the hydration products, should therefore be regarded as tentative and require confirmation by phase-resolved techniques such as XRD and thermogravimetric analysis.

Morphological examination reveals that the reference sample has a more porous structure compared to the samples containing additives. In addition to sponge-like and needle-like structures resembling the C-S-H structure, the presence of more massive, smooth, and calcium hydroxide (CH)-like layered structures was detected. The fact that the structures showing high Ca^2+^ signals in the EDS analysis results are similar suggests that these structures are Portlandite (CH). Morphological examination reveals that these structures occupy a large area on the matrix surface. EDS analyses conducted using the area scan method determined that the atomic Ca/Si ratio is approximately 3.33. Although this ratio varies depending on the age and composition of the cement paste, it is typically around 2 [69]. This situation indicates that the reference sample contains a high proportion of CH, or that the sample has carbonated to some extent, becoming rich in Ca^2+^, or that there are cement particles whose hydration has not yet been completed. The carbon detected in the reference sample is thought to be due to sample coating, contamination, or carbon band reflections.

Morphological examination of the QSM-5 sample reveals that it forms a denser and more homogeneous matrix compared to the reference sample. In addition, structures assessed as C-S-H, observed in needle-like and sponge-like forms, are clearly visible. It is understood that the structures identified as CH in the reference sample occupy less space and are smaller in size in the QSM-5 sample. According to EDS analyses, the carbon content was higher than in the reference sample. This is consistent with the presence of the polysaccharide admixture within the cement paste, although the semi-quantitative nature of EDS does not allow the organic content to be quantified or the formation of a film to be confirmed. Similarly, EDS analyses show that the atomic Ca/Si ratio is approximately 3.09. As with the reference sample, this high Ca/Si ratio indicates a high density of CH structures and suggests possible carbonation and the presence of unhydrated cement particles. However, since all samples were tested under identical conditions, carbonation can be disregarded. The lower Ca/Si ratio obtained for the mucilage-containing samples may indicate an interaction between the mucilage and calcium-rich phases; however, this remains a tentative interpretation. Indeed, similar findings have been reported in the literature in studies conducted with plant mucilages [70].

In the FSM-5 sample, however, the atomic Ca/Si ratio decreased to approximately 2.9. FSM exhibited a mechanism similar to that observed in QSM. The lower Ca/Si ratio in the mucilage-containing samples is consistent with a reduced proportion of calcium-rich phases at the analysed regions, although the present data do not allow the underlying phase changes to be identified. A similar trend was observed for FSM, suggesting comparable behaviour, though the mechanism cannot be confirmed from EDS alone.

It has been reported that polysaccharides and proteins in plant mucilages form carboxyl-containing complexes that bind calcium ions in the cement matrix, and that Portlandite influences crystallization by interacting with mucilage components [70]. Similarly, Ramírez-Arellanes et al. (2012) reported that calcium hydroxide precipitation was reduced with the addition of mucilage [71]. Furthermore, previous studies have reported that polysaccharides manipulate C-S-H and Portlandite formation [66,72]. At the microstructural level, water-soluble polysaccharides have also been reported to modify the morphology of the portlandite crystals and to form polymer bridges between the layered Ca(OH)_2_ platelets, gluing the layers together, increasing interparticle bonding and producing a more cohesive matrix with fewer microcracks [73]. The morphological and compositional features observed in the present SEM-EDS analysis are consistent with these reported mechanisms; as noted above, however, they cannot on their own establish which mechanism operates in the present system, and phase-resolved characterisation would be required to do so.

The carbon signal obtained by EDS cannot be attributed unambiguously to the mucilage: light-element quantification is only semi-quantitative, and the measured carbon may also arise from surface carbonation, chamber contamination or the conductive coating. The elevated carbon in the mucilage-containing pastes should therefore be read as relative organic enrichment rather than a quantitative measure of mucilage content. Similarly, the reduction in the Ca/Si ratio is consistent with Ca^2+^ complexation by mucilage carboxyl groups, but the EDS data alone cannot establish this, as the interaction volume spans several hydration phases. More generally, SEM-EDS yields morphological and elemental information but cannot identify crystalline phases; the C-S-H and portlandite assignments made here rest on morphology and local composition, and phase-resolved techniques such as XRD, together with FTIR or thermogravimetric analysis, would be needed to confirm them. These lie beyond the present scope and are recommended for future work.

## 4. Conclusions

The rheological performance of quince seed mucilage (QSM) and flaxseed mucilage (FSM) as bio-based viscosity-modifying agents was evaluated and compared with welan gum (WG) at equal mass dosages of 0.005–0.1% by weight of cement. The main findings are as follows.

All three admixtures increased the yield stress and plastic viscosity of the cement paste and reduced its flowability with increasing dosage. Yield stress rose from ≈0 Pa for the reference to up to 116.5 Pa for QSM.

The Herschel–Bulkley model described the flow curves better than the Bingham model. The flow behaviour index revealed distinct mechanisms: QSM was strongly shear-thinning at all dosages (*n* ≈ 0.49–0.63), WG was moderately shear-thinning (*n* ≈ 0.69–0.91), and FSM shifted from near-Newtonian to shear-thinning with increasing dosage, indicating a progressively stronger structuring effect.

The practical dosage windows identified were 0.005–0.025% for QSM and 0.025–0.05% for FSM, with optimum performance at 0.01% and 0.05% respectively; above these limits, both mucilages produced mixtures that were no longer practically placeable, whereas WG remained workable throughout. The compressive strength reduction did not exceed 8%. One-way ANOVA detected a significant effect of dosage only for QSM; however, given the small number of replicates, the power to detect differences in this magnitude was limited, and the absence of a detected difference should not be interpreted as evidence of no effect.

These results indicate that both mucilages can act as effective bio-based viscosity-modifying agents within defined dosage ranges.

These distinct behaviours suggest different practical roles: QSM is most appropriate where strong cohesion and segregation resistance are required at low dosage, though within a narrow effective range. WG offers the most gradual and controllable rheological modification over a wide dosage range, suitable for applications requiring tunable control. FSM, with its threshold-like response, is best suited to moderate viscosity modification at higher dosages. The selection among them can therefore be guided by the specific rheological and stability requirements of the application.

### Limitations and Future Work

This study has several limitations that define directions for future work. The admixtures were compared on an equal-mass basis rather than at equivalent viscosity, which is recommended for future studies given that the mucilage extracts are not purified polysaccharides. Rheological measurements were performed on pastes rather than mortars; as the sand fraction was identical in all mixtures, the paste behaviour is taken to reflect that of the mortars, though absolute values may differ between scales. Setting time was assessed only at the highest dosage, so the dose-dependence of retardation remains to be resolved, and dosages below 0.005% warrant further study. Finally, a quantitative cost and life-cycle comparison with commercial viscosity-modifying agents was beyond the present scope and is recommended for future work. Some of the test methods used here, in particular the mini-column segregation test and the vane-in-cup rheometry, are adaptations of established procedures rather than fully standardised methods, and the rheological measurements were performed as single determinations; the absolute values should therefore be regarded as indicative and used for comparison between mixtures rather than as standardised material properties. The superplasticiser dosage was held constant to isolate the independent effect of each VMA. In practical mix design, however, the superplasticiser dosage would typically be adjusted to achieve a target flowability; the present results therefore describe the intrinsic rheological effect of each admixture rather than an optimised mix design, and this should be considered when applying the findings in practice.

## Figures and Tables

**Table 1:** Comparison of the FTIR spectra of quince seed mucilage (QSM) and flaxseed mucilage (FSM).

QSM (cm^−1^)	FSM (cm^−1^)	Assignment
3600–3050	3600–3050	ν(O-H), broad
2925	2925	ν_as(C-H)
2856	2856	ν_s(C-H)
1593	1593	ν_as(COO^−^)
1417	1417	ν_s(COO^−^)
1305	1305	ν(C-O)
1239	1238	ν(C-O)
1177	1177	ν(C-O-C), glycosidic
1106	1106	ν(C-O)
1011	1011	ν(C-O-C), skeletal
825	825	pyranose ring
Peak height ratio	QSM	FSM
A(1593)/A(1011)	2.01 ± 0.41	2.22 ± 0.54
A(O-H)/A(1011)	0.13 ± 0.02	0.66 ± 0.14

*All band positions coincided to within 1 cm^−1^, that is, within the spectral resolution of the measurement. Peak heights were determined from baseline-corrected spectra using linear two-point baselines with anchor points at the local minima on either side of each band. The stated uncertainties correspond to the variation obtained when the baseline anchor points were displaced by ±30 cm^−1^; the spectra were acquired as single measurements, so this range reflects baseline sensitivity rather than measurement repeatability. Because absolute band intensities are not directly comparable between separately measured specimens, only ratios within each spectrum are compared*.

**Table 2:** Prepared mixtures.

Mixture	OPC, kg/m^3^	Sand, kg/m^3^	Water, kg/m^3^	Superplasticizer, % *	WG, % *	QSM, % *	FSM, % *
Ref	600	1437	240	1	-	-	
WG-1	600	1437	240	1	0.005	-	
WG-2	600	1437	240	1	0.01	-	
WG-3	600	1437	240	1	0.025	-	
WG-4	600	1437	240	1	0.05	-	
WG-5	600	1437	240	1	0.1	-	
QSM-1	600	1437	240	1	-	0.005	
QSM-2	600	1437	240	1	-	0.01	
QSM-3	600	1437	240	1	-	0.025	
QSM-4	600	1437	240	1	-	0.05	
QSM-5	600	1437	240	1	-	0.1	
FSM-1	600	1437	240	1	-	-	0.005
FSM-2	600	1437	240	1	-	-	0.01
FSM-3	600	1437	240	1	-	-	0.025
FSM-4	600	1437	240	1	-	-	0.05
FSM-5	600	1437	240	1	-	-	0.1

* By weight of the binder.

**Table 3:** Bingham and Herschel–Bulkley parameters fitted to the descending flow curves of the pastes.

	Bingham			Herschel–Bulkley			
Mix	τ_0_ (Pa)	μ_p_ (Pa·s)	R^2^ₐₑⱼ	τ_0_ (Pa)	K (Pa·s^n^)	*n*	R^2^ₐₑⱼ
Ref	0.0	0.21	0.88	0.21	0.018	1.58	0.99
QSM-1	26.1	0.74	0.97	17.1	3.84	0.63	0.98
QSM-2	62.5	1.08	0.95	40.0	10.76	0.50	0.98
QSM-3	116.5	0.97	0.92	95.5	10.22	0.49	0.94
QSM-4	77.19	1.23	0.94	52.32	11.79	0.50	0.97
QSM-5	88.77	2.08	0.95	48.0	19.0	0.51	0.99
FSM-1	0.0	0.32	0.92	0.45	0.04	1.48	0.99
FSM-2	0.36	0.27	0.98	1.60	0.08	1.26	0.99
FSM-3	3.72	0.49	0.99	3.76	0.48	1.00	0.99
FSM-4	34.0	1.04	0.96	14.64	8.88	0.53	0.99
FSM-5	51.8	1.98	0.99	38.59	5.70	0.76	0.98
WG-1	6.2	0.40	0.99	5.44	0.58	0.91	0.99
WG-2	27.0	0.86	0.98	20.0	2.96	0.72	0.99
WG-3	72.6	1.59	0.97	57.9	6.18	0.69	0.99
WG-4	74.5	2.10	0.98	55.0	8.19	0.69	0.99
WG-5	60.4	2.32	0.98	47.7	7.51	0.73	0.99

## Data Availability

The data presented in this study are available on request from the corresponding author. The data are not publicly available due to a pending patent application related to this work.
